# Predicting 1-, 3-, 5-, and 8-year all-cause mortality in a community-dwelling older adult cohort: relevance for predictive, preventive, and personalized medicine

**DOI:** 10.1007/s13167-023-00342-4

**Published:** 2023-11-03

**Authors:** Yequn Chen, Xiulian Deng, Dong Lin, Peixuan Yang, Shiwan Wu, Xidong Wang, Hui Zhou, Ximin Chen, Xiaochun Wang, Weichai Wu, Kaibing Ke, Wenjia Huang, Xuerui Tan

**Affiliations:** 1 Department of Community Monitoring, First Affiliated Hospital of Shantou University Medical College, Shantou, 515041 Guangdong China; 2 Centre for Precision Health, Edith Cowan University, Perth, WA 6027 Australia; 3 Department of Health Management Centre, First Affiliated Hospital of Shantou University Medical College, Shantou, 515041 Guangdong China; 4 Clinical Research Centre, First Affiliated Hospital of Shantou University Medical College, No. 22 Xinling Road, Jinping District, Shantou, 515041 Guangdong China

**Keywords:** Predictive preventive personalized medicine (PPPM), All-cause mortality, Community-dwelling older adults, Nomogram, Risk factors, Primary care

## Abstract

**Background:**

Population aging is a global public health issue involving increased prevalence of age-related diseases, and concomitant burden on medical resources and the economy. Ninety-two diseases have been identified as age-related, accounting for 51.3% of the global adult disease burden. The economic cost per capita for older people over 60 years is 10 times that of the younger population. From the aspects of predictive, preventive, and personalized medicine (PPPM), developing a risk-prediction model can help identify individuals at high risk for all-cause mortality and provide an opportunity for targeted prevention through personalized intervention at an early stage. However, there is still a lack of predictive models to help community-dwelling older adults do well in healthcare.

**Objectives:**

This study aims to develop an accurate 1-, 3-, 5-, and 8-year all-cause mortality risk-prediction model by using clinical multidimensional variables, and investigate risk factors for 1-, 3-, 5-, and 8-year all-cause mortality in community-dwelling older adults to guide primary prevention.

**Methods:**

This is a two-center cohort study. Inclusion criteria: (1) community-dwelling adult, (2) resided in the districts of Chaonan or Haojiang for more than 6 months in the past 12 months, and (3) completed a health examination. Exclusion criteria: (1) age less than 60 years, (2) more than 30 incomplete variables, (3) no signed informed consent. The primary outcome of the study was all-cause mortality obtained from face-to-face interviews, telephone interviews, and the medical death database from 2012 to 2021. Finally, we enrolled 5085 community-dwelling adults, 60 years and older, who underwent routine health screening in the Chaonan and Haojiang districts, southern China, from 2012 to 2021. Of them, 3091 participants from Chaonan were recruited as the primary training and internal validation study cohort, while 1994 participants from Haojiang were recruited as the external validation cohort. A total of 95 clinical multidimensional variables, including demographics, lifestyle behaviors, symptoms, medical history, family history, physical examination, laboratory tests, and electrocardiogram (ECG) data were collected to identify candidate risk factors and characteristics. Risk factors were identified using least absolute shrinkage and selection operator (LASSO) models and multivariable Cox proportional hazards regression analysis. A nomogram predictive model for 1-, 3-, 5- and 8-year all-cause mortality was constructed. The accuracy and calibration of the nomogram prediction model were assessed using the concordance index (C-index), integrated Brier score (IBS), receiver operating characteristic (ROC), and calibration curves. The clinical validity of the model was assessed using decision curve analysis (DCA).

**Results:**

Nine independent risk factors for 1-, 3-, 5-, and 8-year all-cause mortality were identified, including increased age, male, alcohol status, higher daily liquor consumption, history of cancer, elevated fasting glucose, lower hemoglobin, higher heart rate, and the occurrence of heart block. The acquisition of risk factor criteria is low cost, easily obtained, convenient for clinical application, and provides new insights and targets for the development of personalized prevention and interventions for high-risk individuals. The areas under the curve (AUC) of the nomogram model were 0.767, 0.776, and 0.806, and the C-indexes were 0.765, 0.775, and 0.797, in the training, internal validation, and external validation sets, respectively. The IBS was less than 0.25, which indicates good calibration. Calibration and decision curves showed that the predicted probabilities were in good agreement with the actual probabilities and had good clinical predictive value for PPPM.

**Conclusion:**

The personalized risk prediction model can identify individuals at high risk of all-cause mortality, help offer primary care to prevent all-cause mortality, and provide personalized medical treatment for these high-risk individuals from the PPPM perspective. Strict control of daily liquor consumption, lowering fasting glucose, raising hemoglobin, controlling heart rate, and treatment of heart block could be beneficial for improving survival in elderly populations.

**Supplementary Information:**

The online version contains supplementary material available at 10.1007/s13167-023-00342-4.

## Introduction

### Population aging is an important public health issue

Population aging is a global phenomenon. The number of people over the age of 60 years was 600 million in 2000 and is expected to be 2 billion worldwide in 2050 [1]. Population aging increased the prevalence of age-related diseases and a concomitant burden on medical resources and the economy. Ninety-two diseases have been identified as age-related, accounting for 51.3% of the global adult disease burden [2]. Among individuals over 60 years old, 75.8% have at least one chronic disease, 58.3% have hypertension, 19.4% have diabetes, and 10.5% have hypercholesterolemia, which often reduces the quality of life and life expectancy [3]. The economic cost per capita for older people over 60 years was 10 times that of the younger population [4]. Early intervention may improve prognosis and extend life expectancy for older adults [5]. Current healthcare strategies mainly involve reactive medicine, which is a delayed intervention approach in response to already existing clinical signs and symptoms, and which leads to high cost of most medical services and high long-term mortality [6]. To improve this phenomenon, in the framework of predictive, preventive, personalized medicine, physicians and scientists are trying to transform the paradigm from delayed reactive medicine to proactive medicine by developing risk-prediction models and screening for risk factors in community-dwelling older adults by using clinical multidimensional variables.

### Development of an all-cause mortality prediction model for the elderly in the framework of PPPM

Predictive preventive personalized medicine (PPPM) is an integrative approach that mainly contains three aspects: individual predisposition prediction, targeted preventive measures, and personalized treatment algorithms [7]. The European Association for Predictive, Preventive and Personalized Medicine (EPMA) has proposed the concept of anti-aging that pays much attention to preventive healthcare measures, rather than hospital-based medicine [5]. In view of the aging populations, a PPPM approach in health care systems is critical for keeping the quality of health care on a global scale [8]. Following the concept of PPPM, developing an effective risk-prediction model to recognize high-risk individuals early, providing targeted prevention, and carrying out personalized treatment are important for community-dwelling older adults. However, prior studies mainly focus on hospitalized patients with pronounced symptoms [9–11]. Many community-dwelling older adults suffer from at least one chronic disease, but without obvious symptoms, which leads to delayed diagnosis and personalized treatment. Community-dwelling older adults also often fail to recognize risk factors of long-term mortality and easily ignore proactive prevention management. Demographic variables, comorbid conditions, lifestyle behaviors, laboratory-based molecular patterns, and electrocardiogram indices have been reported to be independently associated with long-term mortality [12–16]. Multidimensional clinical data integrating all these factors may improve the accuracy of predictive models. However, this has not been fully considered. Therefore, we integrated multidimensional clinical data, developed a risk prediction model, and identified risk factors for 1-, 3-, 5-, and 8-year all-cause mortality, providing targeted prevention and individual treatment for community-dwelling older adults, based on the identified risk factors, in the framework of PPPM.

## Working hypothesis

It is hypothesized that an early application PPPM approach would contribute to the healthcare of older adults. Compared to a separate questionnaire, the clinical multidimensional variables add an electrocardiogram (ECG) and clinical laboratory examination, which reflect the characteristics of the participants more comprehensively and objectively. In this study, we predict that an accurate all-cause mortality risk-prediction model can be developed using clinical multidimensional variables for community-dwelling older adults. Such a model would add value to personalized medicine for community-dwelling older adults by enabling PPPM. To test our hypothesis, we developed and externally validated a nomogram prediction model. AUC, C-index, IBS, and calibration curves were used to evaluate the discriminatory and calibration abilities of the nomogram. Decision curve analysis was used to assess the clinical utility of the nomogram.

## Methods

### Participants and sampling methods

A two-center cohort study was established in 2012 by the First Affiliated Hospital of Shantou University Medical College in Shantou, Guangdong Province, southern, China. We used a multi-stage, stratified cluster sampling method, and selected two out of seven districts (counties) in the Shantou City, namely, the Haojiang and Chaonan districts. Forty-five communities in the Chaonan district and thirty-two communities in the Haojiang district were selected. A health census was carried out in these communities for the whole population over 18 years of age. Populations meeting inclusion and exclusion criteria were included in the final analysis. Inclusion criteria: (1) community-dwelling adult, (2) resided in the districts of Chaonan or Haojiang for more than 6 months in the past 12 months, and (3) completed a health examination. Exclusion criteria: (1) age less than 60 years, (2) more than 30 incomplete variables, (3) no signed informed consent. This study was in accordance with the principles of the Declaration of Helsinki and approved by the Ethics Committee of the First Affiliated Hospital of Shantou University Medical College (NO: SDFY-EC-SOP-004–3.0-A06).

#### Data collection

The demographic characteristics, lifestyle behaviors, symptoms, medical history, and family history were collected via a standardized questionnaire by trained medical personnel. The information included age, gender, alcohol status, smoking status, daily liquor consumption, and history of cancer. Physical examinations were performed by trained physicians, who provided patient information including height, weight, waist circumference (WC), hip circumference (HC), waist-to-hip ratio (WHR), and systolic and diastolic blood pressures (SBP; DBP). WC was measured immediately above the iliac crest and HC at the maximal circumference of the buttocks, both in centimeters (cm). WHR was calculated by dividing WC by HC. SBP and DBP were presented as the average of three measurements on the right arm using a sphygmomanometer after resting for at least 10 min. BMI was calculated as weight (kg) divided by the square of the height (cm^2^).

Blood samples were collected from participants who had fasted for at least 10 h overnight and were analyzed within 2 h of collection for multiple indicators, including white blood cell counts (WBC), red blood cell counts (RBC), hemoglobin, percentage of monocytes, percentage of lymphocytes, fasting glucose, high-density lipoprotein (HDL), low-density cholesterol (LDL), and creatinine in the clinical laboratory of the First Affiliated Hospital of Shantou University Medical College. The study strictly followed standard laboratory protocols and procedures.

An electrocardiogram (ECG) examination was conducted using a standard 12-lead ECG (NIHON KOHDEN ECG-1350p), by technical staff. A standard 12-lead ECG, whose frequency response characteristics were in accordance with American Heart Association recommendations, was recorded at 25 mm/s and 0.1 mv/mm standardization during quiet respiration [17]. All ECG parameters, including heart rate, *P*-wave duration (PWD), *R* wave in V5 voltage (RV5), and *S* wave in V1 voltage (SV1) were acquired from the ECG database. Overall, a total of 95 variables were collected for the analysis (Supplementary Material Table s1).

#### Outcome evaluation

Follow-up visits for outcomes were conducted annually over 8 years from 2012 to 2021, including face-to-face interviews and telephone interviews, and death was verified from the medical death database. The primary outcome of the study was all-cause death.

#### Feature selection

We interpolated the data of Chaonan and Haojiang districts by using *R* multivariate imputation, respectively. There was no information leakage between these two districts. Variables with more than 30% missing were deleted without interpolation. The rates of missing data for the Chaonan district are shown in the supplementary material (Table s2). The 3091 individuals from Chaonan were randomly divided into two groups after data interpolation, where 75% (*n* = 2318) of participants were allocated to the training set, and the remaining 25% (*n* = 773) were allocated to the internal validation set. The rates of missing data for Haojiang district are shown in the supplementary material (Table s3). There were 1994 individuals from Haojiang who were recruited as the external validation set. The prediction model was developed using the training set. Feature selection was conducted by least absolute shrinkage and selection operator (LASSO) and multivariable Cox proportional-hazards regression analysis.

#### Model development and evaluation

Based on LASSO-Cox analyses, independent risk features were selected and incorporated into the nomogram prediction model for 1-, 3-, 5-, and 8-year all-cause mortality. The validity of the nomogram prediction model was then assessed through an internal validation set and an external validation set. The two sets shared similar characteristics with the training set, as shown in Table 1 and Supplementary Material Table s4. AUC, C-index, IBS, and the calibration curves were used to evaluate the discriminatory and calibration abilities of the nomogram. Decision curve analysis was performed to assess the clinical usefulness of the nomogram. We calculated the risk scores according to the formula (risk score = coefficient 1 * variable 1 + coefficient *N* * variable *N*). According to the optimum cut-off for risk score, participants were divided into low-risk and high-risk groups [18]. Kaplan–Meier survival curves were used to describe the risk stratification. Finally, to facilitate clinical work, a corresponding web calculator was established to present and help understand the nomogram prediction model.

**Table 1:** Baseline characteristics of the training and internal validation sets (*N* = 3091)

Characteristic	Training set	Internal validation set	*P-*value
Number	2318	773	
End, death, *n* (%)	279 (12.0)	91 (11.8)	0.890
Time, year, median (IQR)	4.25.00 (3.75)	12.00 (12.08)	0.090
Age, years, median (IQR)	66 (9)	66.00 (9)	0.080
Gender, female, *n* (%)	1510 (65.1)	482 (62.4)	0.170
BMI, kg/m^2^, median (IQR)	24.15 (4.51)	23.93 (4.31)	0.790
WHR, median (IQR)	0.91 (0.07)	0.91 (0.07)	0.480
SBP, mmHg, median (IQR)	140 (35)	142.00 (30)	0.210
DBP, mmHg, median (IQR)	86 (15)	88.00 (15)	0.390
Smoking, *n* (%)	689 (29.7)	222 (28.7)	0.628
Liquor, *n* (%)	33 (1.4)	15 (1.9)	0.402
Beer, *n* (%)	7 (0.3)	1 (0.1)	0.688
Alcohol status (%)			0.120
Never	2107 (90.9)	699 (90.4)	
Current	100 (4.3)	45 (5.8)	
Former	111 (4.8)	29 (3.8)	
Anhelation, *n* (%)	83 (3.6)	39 (5.0)	0.090
Sour regurgitation, *n* (%)	260 (11.2)	84 (10.9)	0.840
CHD, *n* (%)	45 (1.9)	10 (1.3)	0.310
Stroke sequelae, *n* (%)	16 (0.7)	6 (0.8)	1.000
History of cancer, *n* (%)	10 (0.4)	4 (0.5)	1.000
Family history of cancer, *n* (%)	5 (0.2)	1 (0.1)	1.000
WBC 10E + 9/L, median (IQR)	7.22 (2.29)	7.17 (2.21)	0.690
Hemoglobin, g/L, median (IQR)	136 (16.75)	137.00 (17)	0.070
Percentage of monocytes (IQR)	5.9 (2.2)	6.00 (2.38)	0.020
Percentage of lymphocytes (IQR)	36.1 (11.1)	35.90 (10.8)	0.540
LDLc, mmol/L, median (IQR)	3.7 (1.23)	3.68 (1.24)	0.410
Creatinine, µmol/L, median (IQR)	90 (26)	92.00 (25.51)	0.250
Fasting glucose, mmol/L, median (IQR)	5.65 (1.21)	5.58 (1.33)	0.410
Heart rate, median (IQR)	73 (15)	74.00 (16)	0.090
Heart block, *n* (%)	122 (5.3)	50 (6.5)	0.240
PWD, ms, median (IQR)	106 (14)	106.0 (16)	0.100
RV5, mv, median (IQR)	1.69 (0.84)	1.69 (0.82)	0.420
SV1, mv, median (IQR)	0.83 (0.56)	0.80 (0.5)	0.330

*BMI* body mass index, *CHD* coronary heart disease, *DBP* diastolic blood pressure, *IQR* interquartile range, *LDLc* low-density lipoprotein cholesterol, *PWD* P wave duration, *RV5* R wave in V5 voltage, *SV1* S wave in *V1* voltage, *SBP* systolic blood pressure, *WHR* waist hip rate, *WBC* white blood cell counts

### Statistical analysis

All statistical analyses were conducted with R software (version 4.1.0; R Foundation for Statistical Computing, Vienna, Austria). The optimum cut-off value for the risk score was calculated by X-tile software version 3.6.1 [18]. The characteristics of all skewed distributed continuous variables are presented as the median and interquartile range (IQR) determined by Mann–Whitney *U* tests. Categorical variables are presented as numbers and percentages, and analyzed by the chi-square test or Fisher’s exact test. Differences with a two-sided* p*-value less than 0.05 were considered statistically significant. The chained equation (MICE) package was used to deal with continuous missing variables [19]. The “glmnet” package was used for the LASSO algorithms [20]. The “survminer” (https://rpkgs.datanovia.com/survminer/) and “survival” packages (https://github.com/therneau/survival) were used for Cox proportional hazard regression analysis and Kaplan–Meier curves [21]. The nomogram and calibrated curve were generated with the “rms” package (https://cran.r-project.org/web/packages/rms/). The web calculator was developed using the “DynNom” package [22].

## Results

### Baseline characteristics of participants

**Fig. 1:**
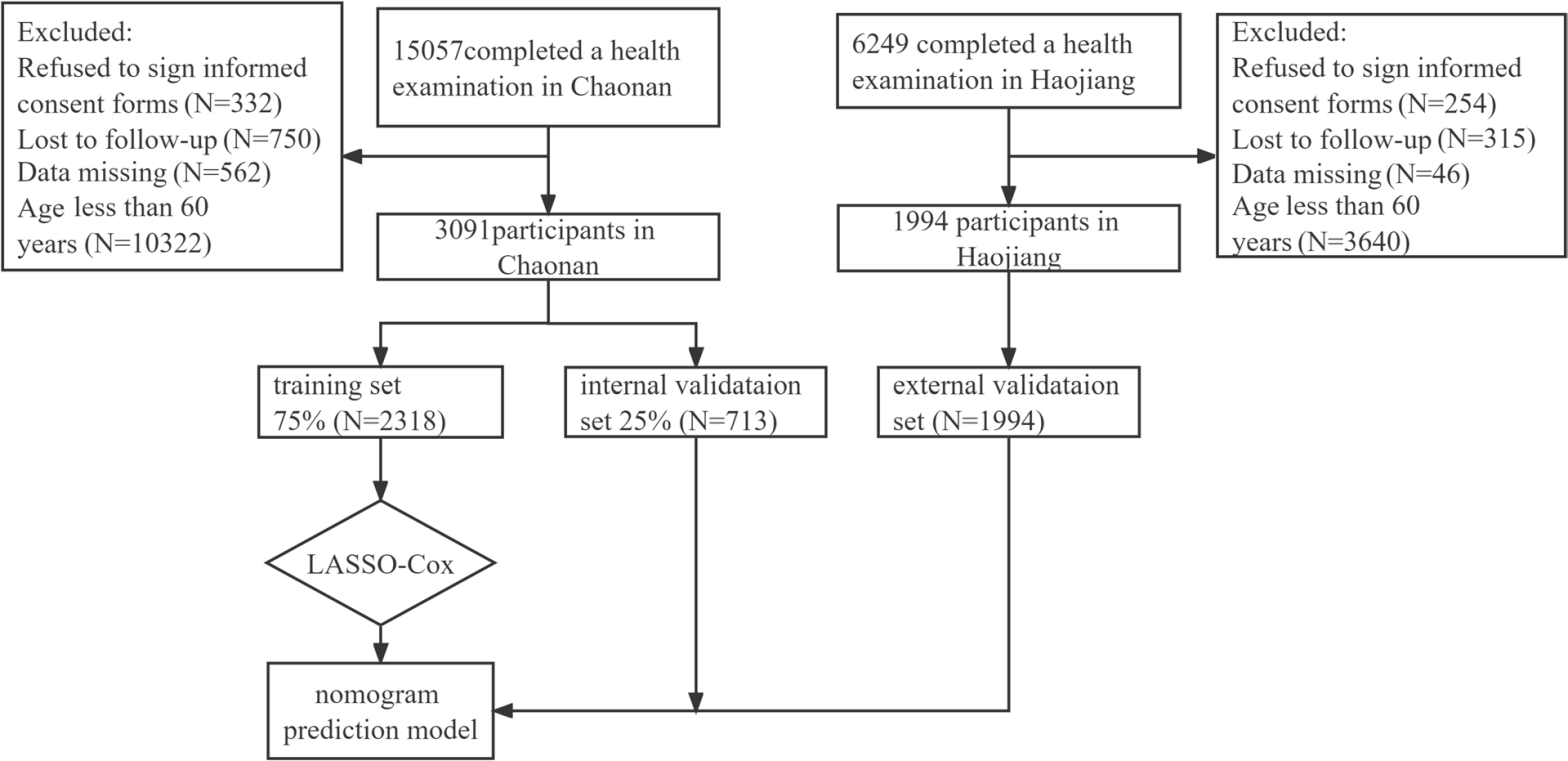
Flowchart of participants included in this cohort study

There were 15,057 participants in Chaonan who completed the health examination. Excluded subjects were 332 participants who refused to sign the informed consent form, 750 participants who dropped out of follow-up, 562 participants who had more than 30 data variables missing, and 10,322 participants who were younger than 60 years old. The 3091 participants from Chaonan were included in the training and internal validation sets. Another 1994 individuals from Haojiang were used for the external validation set. Ultimately, a total of 5085 participants were included in the final analysis and followed up for 16,432 person years (Fig. 1). The baseline characteristics of the participants in the training and internal validation sets are shown in Table 1. All-cause death occurred in 370 of the 3091 Chaonan participants during follow-up (11.97%, 192 males, and 178 females). All-cause death occurred in 138 of 1994 Haojiang residents in the external validation set (6.92%, 75 males and 63 females). The baseline characteristics of the external validation set are shown in Supplementary Material Table s2.

### Feature selection

LASSO was performed on the training set (*N* = 2318, variables = 95). From the LASSO coefficient profiles of the 95 variables and tenfold cross-validation for tuning parameter selection in the LASSO model, twenty-five features were selected from the training set: age, gender, BMI, anhelation, sour regurgitation, smoking status, alcohol status, daily beer consumption, daily liquor consumption, stroke sequelae, WBC, hemoglobin, percentage of monocytes, percentage of lymphocytes, fasting glucose, LDL, creatinine, coronary heart disease, history of cancer, family history of cancer, heart rate, heart block, RV5, SV1, and PWD (Fig s1a and b). In the multivariable Cox proportional-hazards regression analysis, nine potential predictors were selected based on the hazard ratios (95% CI) and *p-*values. The nine predictive factors that were selected involved two demographic characteristics (age and gender), two lifestyle behavioral features (alcohol status and daily liquor consumption), one medical history feature (history of cancer), two laboratory-based measures (fasting glucose and hemoglobin), and two ECG parameters (heart rate and heart block) (Table 2). For the training set, The LASSO-Cox AUC and C-index were 0.793 (95% CI 0.765–0.822) and 0.792 (95% CI 0.763–0.821), respectively. For the internal validation set, the corresponding results were 0.800 (95% CI 0.751–0.849) and 0.828 (95% CI 0.785–0.871). We found good agreement between the AUC and C-index. The IBSs of these two sets were less than 0.25, which demonstrated their good calibration (Supplementary Material Table s5).

**Table 2:** Multivariable Cox proportional-hazards regression analysis of risk factors associated with all-cause mortality in community-based older adults

Variable	Coefficient	HR (95% CI)	*P-*value
Age	0.084	1.088 (1.069–1.107)	< 0.001
Gender			
Male	Reference		
Female	− 0.431	0.65 (0.469–0.901)	0.010
Daily liquor consumption	0.007	1.007 (1.003–1.012)	< 0.001
Hemoglobin	− 0.018	0.982 (0.973–0.992)	< 0.001
Fasting glucose	0.073	1.075 (1.018–1.136)	0.009
Heart rate	0.009	1.009 (1.000–1.018)	0.045
History of cancer			
No	Reference		
Yes	1.342	3.828 (1.202–12.196)	0.023
Heart block			
No	Reference		
Yes	0.462	1.587 (1.012–2.487)	0.044
Alcohol status			
Never	Reference		
Current	0.27	1.31(0.763–0.764)	0.330
Former	0.477	1.611 (1.078–2.407)	0.020

### Nomogram prediction model

**Fig. 2:**
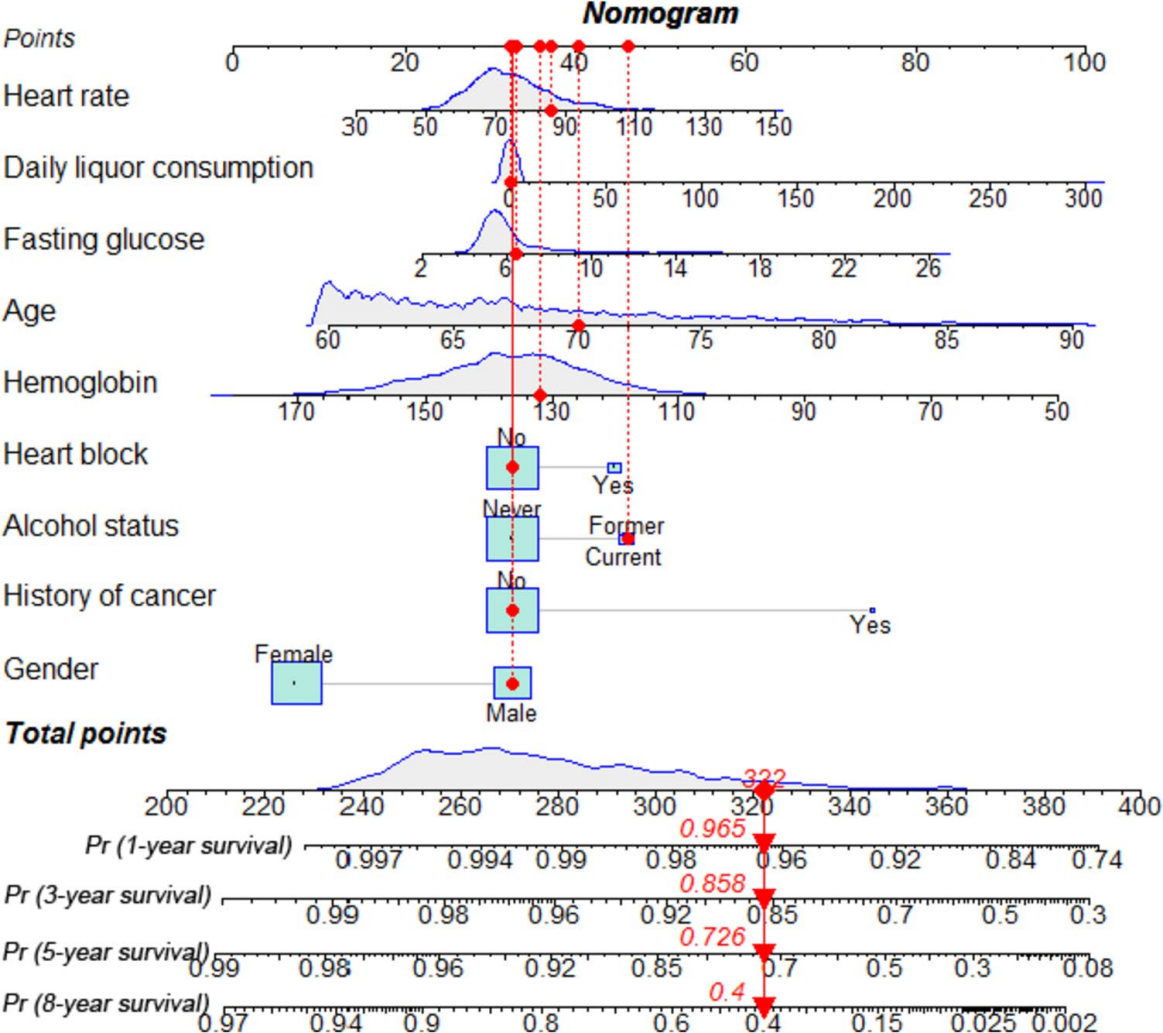
Nomogram for predicting 1-, 3-, 5-, and 8-year mortality in community-dwelling older adults. The corresponding value is found on each variable axis of the nomogram, and then a red line and dots are drawn upward to determine the points received by each variable, the sum of these points is located on the total points axis, and a line is drawn downward to the survival axes to determine the probability of 1-, 3-, 5-, and 8-year mortality in community-dwelling older adults. For instance, if a patient was a 70-year-old male, with no history of cancer, who formerly drank alcohol, and had a current liquor consumption of 0 ml/day, hemoglobin 132 g/L, glucose 6.43 mmol/L, heart rate 86 beats/minute, and no heart block, he would have a total of 322 points. This participant would be predicted to have an approximately 96.5% probability of survival at 1 year, 85.8% probability of survival at 3 years, 72.6% probability of survival at 5 years, and 40% probability of survival at 8 years

**Fig. 3:**
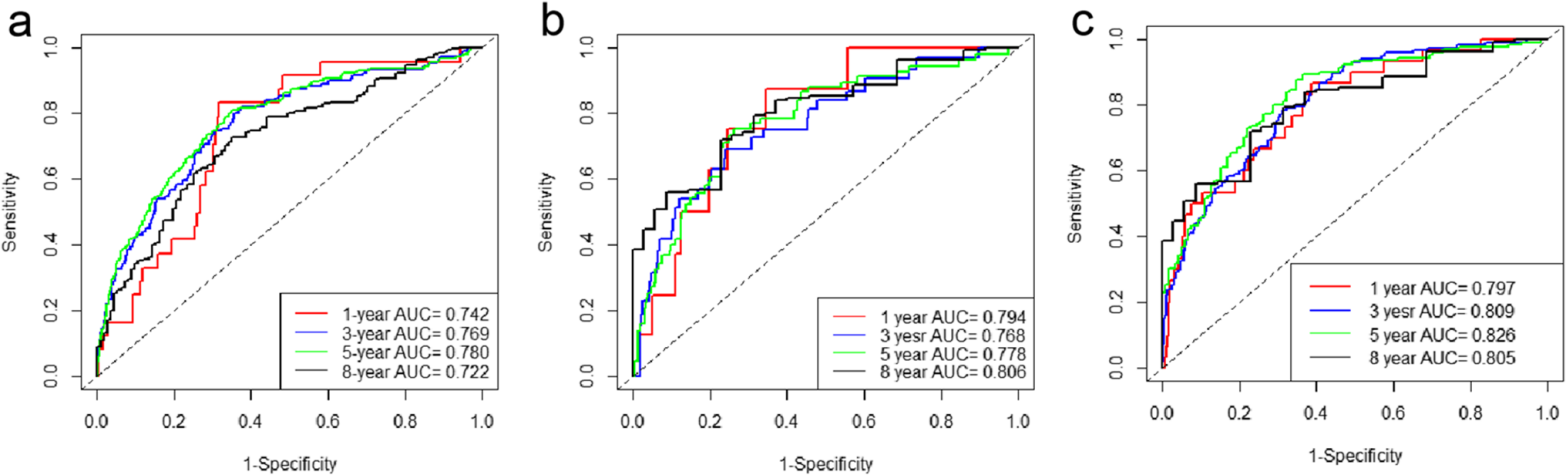
ROC curves of the nomogram for 1-, 3-, 5-, and 8-year all-cause mortality in a community-dwelling older adult cohort in the training set (**a**), internal validation set (**b**), and external validation set (**c**). **a** In the training set, the AUCs of the nomogram for 1-, 3-, 5-, and 8-year all-cause mortality were 0.741, 0.769, 0.780, and 0.722, respectively. **b** In the internal validation set, the AUCs of the nomogram for 1-, 3-, 5-, and 8-year all-cause mortality were 0.794, 0.768, 0.778, and 0.806, respectively. **c** In the external validation set, the AUCs of the nomogram for 1-, 2-, 3-, and 4-year all-cause mortality were 0.803, 0.797, 0.809, and 0.751, respectively

**Fig. 4:**
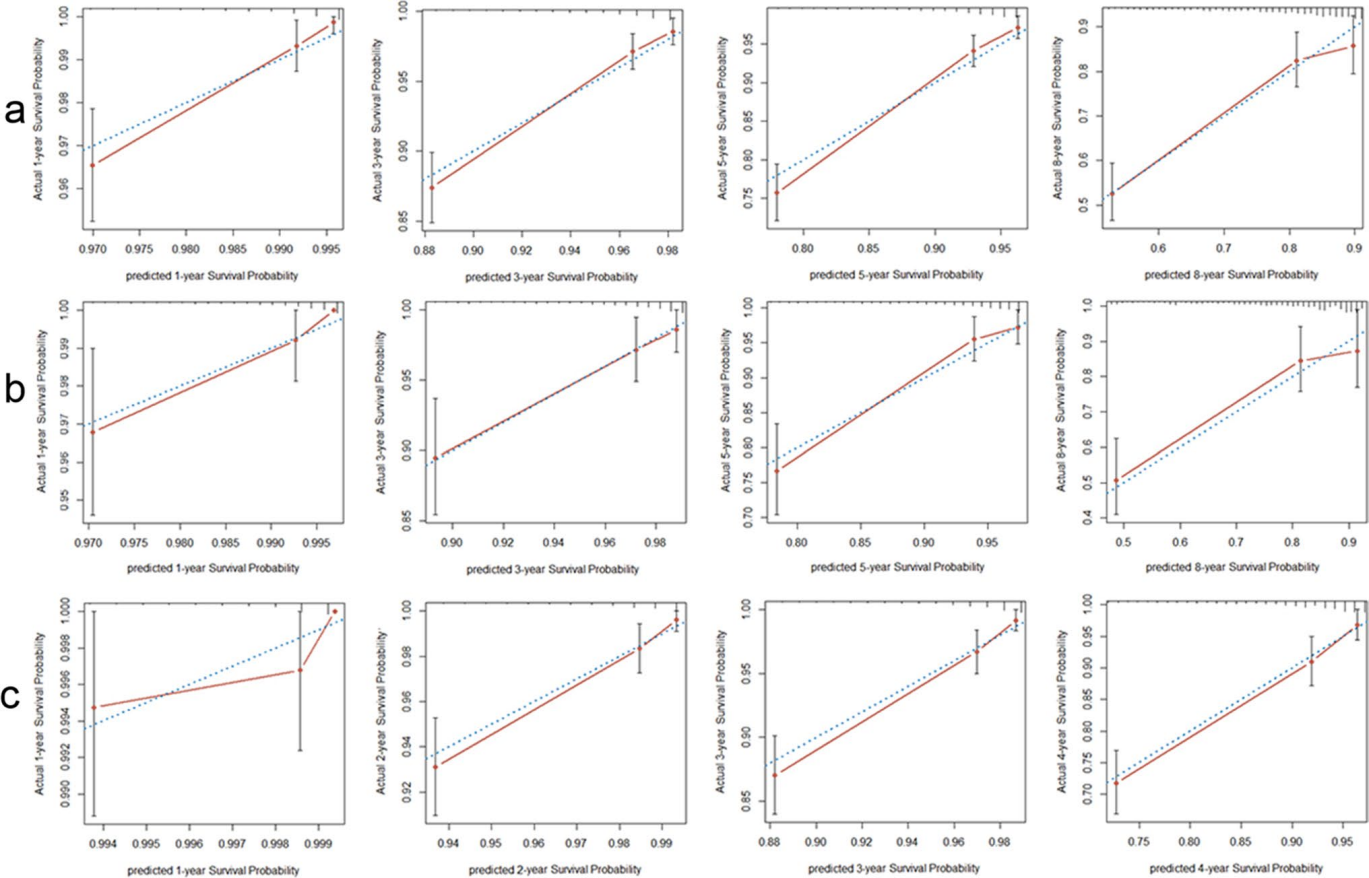
Calibration curves showing how close the nomogram-predicted probability is to the actual probability regarding predicting all-cause mortality in the training set (**a**), internal validation set (**b**), and external validation set (**c**). **a** Calibration curves for 1-, 3-, 5-, and 8-year survival probability for community-based older residents in the training set. **b** Calibration curves for 1-, 3-, 5-, and 8-year survival probability for community-based older residents in the internal validation set. **c** Calibration curves for 1-, 2-, 3-, and 4-year survival probability for community-based older residents in the external validation set. The X-axis is the nomogram-predicted probability; the Y-axis is the observed probability. The dashed blue line represents perfect prediction. The solid red line represents the nomogram performance. The solid red lines above or below the blue dashed line represent an underestimation or overestimation, respectively. The closer the solid red line is to the dashed blue line, the better the prediction by the nomogram

**Fig. 5:**
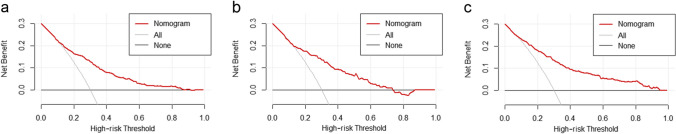
Decision curve analysis of the nomogram for all-cause mortality in the training set (**a**), internal validation set (**b**), and external validation set (**c**). The black line represents the net benefit when no participant was considered to exhibit death, while the gray line represents the net benefit when all participants were considered to exhibit death. The area among the model curve, “non-treated line” (black line) and “treated line” (gray line), represents the clinical usefulness of the model. The further the nomogram model curve is from these two lines (the grey and black lines), the higher the clinical value

As shown in Fig. 2, a nomogram prediction model for predicting 1-, 3-, 5-, and 8-year all-cause mortality among community-dwelling older adults was developed based on the nine potential predictors. Each predictor was assigned a weighted value, and the total points were calculated from the sum of all potential predictors in the nomogram corresponding to a probability of survival at different times. For the training set, internal validation set, and external validation set, the nomogram AUC was 0.767 (95% CI 0.736–0.798), 0.776 (95% CI 0.722–0.831), and 0.806 (95% CI 0.768–0.845); the C index was 0.765 (95% CI 0.733–0.797), 0.775 (95% CI 0.719 –0.831), and 0.797 (95% CI 0.76–0.834); and the IBS was 0.079 (95% CI 0.037–0.123), 0.076 (95% CI 0.045–0.108), and 0.047 (95% CI 0.012–0.083), respectively (Table 3). The AUC and C-index were in good agreement, suggesting that the nomogram model has high predictive ability. The IBSs were less than 0.25, which demonstrated their good calibration. In the training cohort, the AUC values of the nomogram model for 1-, 3-, 5-, and 8-year all-cause mortality were 0.741, 0.769, 0.780, and 0.722, respectively. In both the internal and external validation sets, the AUC values at different time points were greater than 0.750, indicating that the model has good predictive capability (Fig. 3). The calibration curves showed different survival probabilities, predicted by the nomogram, and actual survival (Fig. 4). These results demonstrate that the nomogram prediction model had good predictive and good calibration capabilities. The decision curve analysis (DCA) of the nomogram is shown in Fig. 5. For a given probability threshold, the greater the net benefit of the model, the better. The DCA showed that the use of the nomogram could have a positive net benefit. To facilitate clinical work, a corresponding web calculator was established to present and help understand the prediction model (https://sdfysqjc2021.shinyapps.io/DynNomapp/).

**Table 3:** Discrimination and calibration of the nomogram model at predicting all-cause mortality

	Training set	Internal validation set	External validation set
AUC (95% CI)	0.767 (95% CI 0.736–0.798)	0.776 (95% CI 0.722–0.831)	0.806 (95% CI 0.768–0.845)
C-index (95% CI)	0.765 (95% CI 0.733–0.797)	0.775 (95% CI 0.719–0.831)	0.797 (95% CI 0.76–0.834)
IBS (95% CI)	0.079 (95% CI 0.037–0.123)	0.076 (95% CI 0.045–0.108)	0.047 (95% CI 0.012–0.083)

*AUC* area under curve, *C-index* concordance index, *IBS* integrated Brier score

### Risk stratification

Based on the LASSO-Cox model, we calculated the risk score as follows: Risk score = 0.084 * age − 0.431 * gender (female) + 0.477 * alcohol status (former) + 0.007 * daily liquor consumption − 0.018 * hemoglobin + 0.073 * fasting glucose + 1.342 * history of cancer + 0.462 * heart block + 0.009 * heart rate. The risk score of participants ranged from 2.1 to 7.3, and the optimal cut-off was 4.7. According to the optimum cut-off, people were divided into a low-risk group (risk score ≤ 4.7) and a high-risk group (risk score > 4.7) (Supplementary Material Fig s2). Kaplan–Meier curves showed that the low-risk group exhibited higher survival than the high-risk group both in the training, internal validation, and external validation sets (Supplementary Material Fig s3).

## Discussion

### Summary of the important findings

In this cohort study, we adopted an integrated PPPM approach using multidimensional data to develop and validate a personalized risk model predicting 1-, 3-, 5-, and 8-year all-cause mortality for community-dwelling older adults. Nine risk factors were identified, i.e., increased age, male, alcohol status, higher daily liquor consumption, history of cancer, elevated fasting glucose, lower hemoglobin, higher heart rate, and the occurrence of heart block. The AUC and C-index show high precision of the nomogram model. The lower IBS and calibration curves suggest that the nomogram prediction model exhibits good calibration. Moreover, DCA suggests that the nomogram has a positive net benefit. Our model provides accurate predictions within diverse community-based elderly populations, aiding in the identification of individuals at high risk of mortality and facilitating early interventions for mortality prevention. This model holds significant potential for influencing healthcare and policy formulation for the elderly, fostering the development of personalized preventive and interventional strategies. Our estimations serve as valuable tools in clinical discussions, assisting in informed decision-making, with an emphasis on considering life expectancy in treatment decisions [23]. Through this research, our aim is to propel the shift of healthcare models from traditional treatments towards a more proactive and preventive approach, ultimately enhancing the quality of life for the elderly and reducing all-cause mortality.

### An all-cause mortality prediction model for the elderly

This model holds tremendous potential in the realms of prediction, prevention, and personalized medicine for the elderly, particularly as the global elderly population continues to grow, posing challenges to healthcare resources and economic burdens. Prior research mainly focuses on exploring the progress and prognosis of the disease to reduce mortality [24, 25]. However, few studies have predicted all-cause mortality in community-dwelling older adults. For these hospitalized elderly patients who have significant symptoms, the current healthcare outcomes are considered inadequate. Compared with previous reactive medicine, PPPM is a new integrative paradigm that focuses on predicting and preventing disease before the onset of symptoms, as well as providing personalized treatment and focuses on proactive preventative measures [7]. All-cause mortality prediction, targeted prevention, and intervention before the onset of symptoms are important and could improve the quality of life and extend life expectancy. Previous all-cause mortality prediction models for community populations have been developed in Europe, but their prediction indicators are limited in demographic variables, comorbid conditions, and lifestyle behaviors [26–29]. The participants of these studies were not limited to community-dwelling older adults, and also included young and middle-aged adults, and none of these models underwent external validation [26–29]. There is especially a lack of studies to predict all-cause mortality for community-dwelling older adults in China. To solve this problem, we developed an all-cause mortality risk prediction model in community-dwelling older adults to identify individuals on the basis of individual heterogeneity following the concept of PPPM. To the best of our knowledge, this is the first study using clinical multidimensional variables to develop and validate a nomogram prediction model, with external validation, for 1-, 3-, 5-, and 8-year all-cause mortality among community-dwelling older adults. Our study fills a gap, in the framework of PPPM, for all-cause mortality prediction with multidimensional data and external validation to provide targets for proactive prevention and individual management in personalized medicine of community-dwelling older adults. This easy-to-use prediction model can easily identify elderly people at high risk of all-cause mortality, guide-targeted prevention, provide personalized interventions for high-risk individuals, and promote a paradigm shift from delayed reactive medicine to proactive medicine. Our risk-prediction model can help generate a tailored, targeted PPPM approach that benefits both individuals and healthcare systems.

### Risk factors and targets provide insights for prediction prevention and intervention

Our prediction model helps identify people at high mortality risk for all-cause mortality. However, early prediction may have little effect if effective interventions are not implemented. In this direction, we identified nine risk factors from clinical multidimensional data, including three nonmodifiable (increased age, male, and history of cancer) and six modifiable (alcohol status, daily liquor consumption, elevated fasting glucose, lower hemoglobin, higher heart rate, and the occurrence of heart block) risk factors. The identified risk factors are common and easily obtained in clinical work at low cost, which makes it convenient to use in early personal prevention and interventions for high-risk individuals.

Although the three nonmodifiable risk factors cannot be used for prevention and intervention, there is an important role in prediction for the identification of people at high risk of all-cause mortality. Our study found that increased age was an independent risk factor for all-cause mortality, which is consistent with the previous models [28, 29]. Advanced age may lead to multi-system functional decline, including the immune system, and cardiovascular, metabolic, autoimmune, and neurodegenerative diseases [30], thereby increasing the risk of all-cause mortality. Healthcare professionals should prioritize assessing all-cause mortality risk in elderly patients and implement interventions, such as health assessments, chronic disease management, and promotion of healthy lifestyles, to enhance longevity and quality of life. In our study, male gender had a higher risk of all-cause mortality, which is consistent with the previous models [28, 29]. This gender difference may be attributable in part to differences in lifestyle and behavioral risk factors (e.g. inadequate diet, physical inactivity, tobacco use, and excessive alcohol use), and psychosocial and environmental exposures [31]. Identifying male gender as a risk factor for all-cause mortality helps healthcare professionals to better identify high-risk individuals. It also provides a basis for developing gender-specific interventions to reduce all-cause mortality. We also found that a history of cancer is an independent risk factor for all-cause mortality, which is consistent with the previous studies [28]. Psychosocial stress of cancer survivors may lead to dysregulated immune function and induce chronic inflammation [32]. In addition, some cancer treatments may be cardiotoxic, resulting in cardiovascular disease being the second leading cause of morbidity and mortality in cancer survivors, after recurrent malignancies [33]. Healthcare professionals should pay more attention to all-cause mortality risks for the elderly with a history of cancer. The health and psychosocial stress of cancer survivors should be regularly assessed, and interventions should be made at an early stage to improve their quality of life and extend their life expectancy.

The six modifiable risk factors are beneficial for providing targets for prediction, prevention, and intervention at early stages in high-risk individuals. Heavy alcohol consumption is associated with the risk of fall [34] and cardiovascular disease [35], potentially increasing the cost of unnecessary hospitalization. In our study, we found that high, daily liquor consumption and alcohol status were predictors of all-cause mortality. However, the relationship between alcohol consumption and all-cause mortality may be complex. Continued heavy alcohol use can cause substantial morbidity and mortality from all causes, cancer, and accidents [36]. Light and moderate alcohol consumption have been inversely associated with mortality from all causes, cardiovascular disease, chronic lower respiratory tract diseases, Alzheimer’s disease, and influenza and pneumonia [37]. Clarifying the relationship between alcohol status and higher daily liquor consumption and all-cause mortality can help healthcare professionals better advise their patients. Strict control of daily liquor consumption is a good behavior that will avoid unnecessary hospitalizations and long-term care.

Elevated fasting glucose, whether incident, persistent, or due to diabetes, confers a higher risk of mortality [38]. For patients with diabetes mellitus, a good control of glucose facilitates the reduction of short- and long-term complications, reduces the burden of treatment, and improves quality of life [39]. Our findings emphasize the potential impact of high fasting glucose levels to increase all-cause mortality in older adults. Hyperglycemic states may trigger several physiological and metabolic changes, such as inflammation, oxidative stress, and vascular dysfunction, which may contribute to the onset and progression of multiple chronic diseases [40]. Healthcare professionals should closely monitor blood glucose levels in older patients and intervene as necessary to reduce long-term complications and mortality. This may include maintaining normal blood glucose levels through an improved diet, increased physical activity, medication, or other interventions.

Low hemoglobin, an indicator of anemia, is very common in the older population and is associated with reduced quality of life and increased mortality [41]. It is also strongly associated with more frequent hospitalization and longer hospital stays [42]. Even mild anemia may substantially affect physical and cognitive capacities and quality of life [42]. In our study, a low hemoglobin level was clearly identified as an independent factor associated with all-cause mortality, which is consistent with the previous studies [43]. Low hemoglobin levels may influence the risk of all-cause mortality through mechanisms that reduce oxygen supply, increase cardiovascular burden, and impair immune function. Clinicians should pay special attention to hemoglobin levels in elderly patients and take the necessary steps to correct the low hemoglobin state, including complementary therapies (iron, vitamin B12 or folic acid supplementation, use of erythropoietic drugs) [42] or elimination of the underlying cause to correct the anemia, thereby improving oxygen supply and reducing the risk of all-cause mortality.

Higher heart rates have been reported to be associated with an increased risk of dementia and accelerated rates of cognitive decline in the general elderly population, which can affect the quality of life in older adults [44]. Our findings found that high heart rate is an important risk factor in the increased risk of all-cause mortality in older adults. Previous studies also indicated that accelerated heart rate increases the risk of sudden cardiac death, heart failure, and all-cause mortality [45]. Higher heart rates in patients with stable coronary heart disease raise the risk of complications, even when other factors are under control [46]. Keeping the heart rate of the elderly in the normal range contributes to improved quality of life and survival rates. Clinicians should closely monitor heart rate levels in older patients and take the necessary measures to control high heart rates. This may include heart rhythm medication, physical activity, and measures to improve cardiovascular health.

Our findings show that heart block is associated with a risk of all-cause mortality in older adults. In this study, the heart block includes an atrioventricular block, right bundle branch block, and left bundle branch block. An atrioventricular block is associated with heart failure, atrial fibrillation, and all-cause mortality [47]. Patients with right bundle branch block are more likely to have congestive heart failure, cardiogenic shock, hypertension, and ventricular tachyarrhythmias [48]. The left bundle branch block reduces left ventricular ejection fraction, which is an unfavorable prognostic parameter in patients with congestive heart failure [49]. Heart block may decrease the heart’s perfusion, systolic and diastolic function, and hemodynamics [50]. These lead to an increased risk of cardiac events and thus an increased risk of all-cause mortality. Clinicians should closely monitor the function of the cardiac conduction system in older patients and take the necessary measures to treat heart blocks. This may include medication, pacemaker implantation, and other cardiac therapeutic interventions.

Therefore, the application of the nine risk factors in predicting all-cause mortality among community-dwelling older adults seems to be well-founded. From the perspective of PPPM, modifiable risk factors should be monitored when developing targeted prevention and intervention measures to decrease mortality for community-dwelling older adults. We recommend increased monitoring of alcohol status, daily alcohol consumption, fasting glucose, hemoglobin, and electrocardiograms among older people in the community. Conducting person-specific preventative and treatment measures (e.g. strict control of daily liquor consumption, lowering fasting glucose, raising hemoglobin, controlling heart rate, and treatment of heart block) for high-risk elderly may help improve the quality of life and survival rates [23]. Eliminating these risk factors may reduce the frequency of hospitalization and care, reducing the length of hospital stay and saving healthcare costs. This easy-to-use predictive model may also be able to improve adherence/willingness to health care for the elderly [23]. Moreover, our findings aid in guiding physicians’ decisions on treatment and management, avoiding over- and under-treatment.

### Strengths and limitations

The strengths of the study need to be emphasized: Firstly, in this two-center study, we developed and externally validated a nomogram prediction model, and showed that the model has good discrimination and calibration capabilities. Secondly, this is a clinical multidimensional variable cohort study with a large sample size. The 8 years of follow-up comprised a total of 16,432 person years among 5085 participants. We screened predictors from 95 clinical multidimensional variables, including demographics, lifestyle behaviors, symptoms, medical history, family history, physical examination, laboratory examination, and ECG data. Finally, the prediction models help to predict all-cause mortality in older adults and to identify high-risk individuals. The identified risk factors help the high-risk individuals to take personalized preventive and intervention measures at an early stage. The results of this study have significant clinical value in improving the health status, quality of life, and survival of older people.

There are also several limitations in this study. Firstly, this study lacks certain social factors, for example, health domains, housing status, transportation, years of retirement, or even activities of daily living, as no relevant data was available in the questionnaire. Secondly, we also did not obtain molecular omics data, such as genomics, proteomics, and transcriptome data. Molecular omics testing is mostly used in scientific research and is rarely performed for health checkups. Increasing these social factors and molecular omics data has the potential to improve the accuracy of the model. Finally, the demographic variables of this study were self-reported, which may have resulted in subjective bias, and may cast doubt on model effectiveness. However, the questionnaire was collected by trained medical personnel, which may lower this bias. The final prediction model did not include subjective variables.

## Conclusions and expert recommendations

In conclusion, based on clinical multidimensional variables, we developed and externally validated a personalized prediction model to evaluate 1-, 3-, 5-, and 8-year all-cause mortality for community-dwelling older adults, and offer primary prevention and personalized medical treatment strategies based on modifiable risk factors. Strict control of daily liquor consumption, lowering fasting glucose, raising hemoglobin, controlling heart rate, and treatment of heart blocks will be beneficial for improving survival in elderly populations.

Our study supports the application of PPPM, from different perspectives, in the medical services of all-cause mortality community-dwelling older adults. Firstly, from the perspective of all-cause mortality prediction, we constructed an all-cause mortality risk-prediction model applicable to community-dwelling older adults. The risk-prediction model could identify individuals at high risk of all-cause mortality and contribute to the paradigm shift from delayed reactive medicine to proactive medicine, which could help save public health resources and decrease the mortality of community-dwelling older adults. Secondly, from the perspective of all-cause mortality prevention, current prediction indicators of mortality prediction models are limited in demographic variables, comorbid conditions, and lifestyle behaviors. In this study, we identified risk factors by clinical multidimensional variables, and these risk factors are easy to obtain, thus helping us to monitor the progression of all-cause mortality. Community older adults can prevent all-cause mortality by targeting daily health care against modifiable risk factors. Finally, from the perspective of personalized interventions, clinical multidimensional variables are more comprehensive and objective. Our risk-prediction model can predict the risk of mortality for community-dwelling older adults at different times, and develop tailored interventions through modifiable risk factors to reduce mortality.

### Supplementary Information

Below is the link to the electronic supplementary material.

Supplementary file1 (DOC 815 KB)

Supplementary file2 (DOC 38 KB)

## Data Availability

The data used to support the findings of this study are available from the corresponding author upon reasonable request.
