# Supplementary material for: Predicting 1-, 3-, 5-, and 8-year all-cause mortality in a community-dwelling older adult cohort: relevance for predictive, preventive, and personalized medicine
Source: EPMA J. 2023 Nov 3;14(4):713–26. doi: 10.1007/s13167-023-00342-4 (PMC10713970; doi:10.1007/s13167-023-00342-4)
Supplement: Supplementary file 1 — Supplementary file1 (DOC 815 KB) [file 13167_2023_342_MOESM1_ESM.doc]

**Table s1 The 95 variables included in the analysis**

| Multidimensional variables |  |
| --- | --- |
| Demographic characteristics | |
|  | Age |
|  | Gender |
|  | Marriage |
|  | Profession |
|  | Education |
| Physical examination | |
|  | BMI |
|  | WHR |
|  | SBP |
|  | DBP |
| Lifestyle behaviors | |
|  | Meat |
|  | Viscera |
|  | Vegetable |
|  | Fish |
|  | Curing food |
|  | Vegetable oil |
|  | Animal oil |
|  | Passive smoking |
|  | Smoking status |
|  | Alcohol status |
|  | Tea status |
|  | Smoking consumption |
|  | Wine consumption |
|  | Liquor consumption |
|  | Rice wine consumption |
|  | Beer consumption |
|  | Green tea consumption |
|  | Black tea consumption |
|  | Oolong consumption |
| Symptoms | |
|  | Chest pain |
|  | Anhelation |
|  | Palpitation |
|  | Cough |
|  | Polydipsia |
|  | Syncope |
|  | Dizziness |
|  | Weak |
|  | Urinary sign |
|  | Sour regurgitation |
|  | Stomachache |
| Medical history | |
|  | Hypertension |
|  | Diabetes |
|  | CHD |
|  | Arrhythmia |
|  | Hyperlipidemia |
|  | Stroke |
|  | Stroke of sequelae |
|  | Cancer |
| Family history | |
|  | Family history of hypertension |
|  | Family history of diabetes |
|  | Family history of stroke |
|  | Family history of cancer |
| Laboratory examination | |
|  | WBC |
|  | RBC |
|  | Percentage of monocytes |
|  | Monocytes |
|  | Percentage of lymphocyte |
|  | Lymphocyte |
|  | Eosinophil |
|  | Percentage of eosinophil |
|  | Percentage of neutrophilic |
|  | Neutrophilic |
|  | Percentage of basophil |
|  | Basophil |
|  | RDWCV |
|  | MCV |
|  | MCH |
|  | MCHC |
|  | HCT |
|  | Hemoglobin |
|  | MPV |
|  | Plateletcrit |
|  | Platelet |
|  | TG |
|  | TC |
|  | LDLc |
|  | HDLc |
|  | ALT |
|  | AST |
|  | UA |
|  | Creatinine |
|  | Fasting glucose |
| ECG examination | |
|  | PWD |
|  | [PR interval](#/javascript:;) |
|  | QT interval |
|  | QTc interval |
|  | QRS interval |
|  | QRS-axis |
|  | RV5 |
|  | SV1 |
|  | AF |
|  | Left ventricular high voltage |
|  | Heart rate |
|  | Heart block |
|  | Heart transposition |
|  | ST change |

Abbreviations: ALT, alanine transaminase; AST, aspartate aminotransferase; AF, atrial fibrillation; BMI, body mass index; CHD, coronary heart disease; DBP, [diastolic](../../../../C:/Program%20Files%20(x86)/Youdao/Dict/8.9.9.0/resultui/html/index.html#/javascript:;) [blood](../../../../C:/Program%20Files%20(x86)/Youdao/Dict/8.9.9.0/resultui/html/index.html#/javascript:;) [pressure](../../../../C:/Program%20Files%20(x86)/Youdao/Dict/8.9.9.0/resultui/html/index.html#/javascript:;); HCT, hematocrit; HDLc, high-density lipoprotein cholesterol; LDLc, [low-density lipoprotein](../../../../C:/Program%20Files%20(x86)/Youdao/Dict/8.9.9.0/resultui/html/index.html#/javascript:;) cholesterol; MCV, mean corpuscular volume; MCH, mean corpuscular hemoglobin; MCHC, mean corpuscular hemoglobin concentration; MPV, mean platelet volume; PWD, duration of P wave; RBC, red blood cell; RDWCV, red blood cell volume distribution width; RV5, R wave in V5 voltage; SV1, S wave in lead V1voltage; SBP, systolic blood pressure; TG, triglycerides; TC, total cholesterol; UA, [uric](../../../../C:/Program%20Files%20(x86)/Youdao/Dict/8.9.9.0/resultui/html/index.html#/javascript:;) [acid](../../../../C:/Program%20Files%20(x86)/Youdao/Dict/8.9.9.0/resultui/html/index.html#/javascript:;); WBC, [white](../../../../C:/Program%20Files%20(x86)/Youdao/Dict/8.9.9.0/resultui/html/index.html#/javascript:;) [blood](../../../../C:/Program%20Files%20(x86)/Youdao/Dict/8.9.9.0/resultui/html/index.html#/javascript:;) [cell](../../../../C:/Program%20Files%20(x86)/Youdao/Dict/8.9.9.0/resultui/html/index.html#/javascript:;) counts；WHR, waist hip rate

**Table s2 The 95 variables and the missing rates in the Chaonan group**

| **No.** | **variable** | **Number** | **Missing rates** |
| --- | --- | --- | --- |
| Demographic characteristics | | | |
| 1 | Age | 0 | 0% |
| 2 | Gender | 0 | 0% |
| 3 | Marriage | 12 | 0.39% |
| 4 | Profession | 10 | 0.32% |
| 5 | Education | 10 | 0.32% |
| Physical examination | | | |
| 6 | BMI | 6 | 0.19% |
| 7 | WHR | 11 | 0.36% |
| 8 | SBP | 4 | 0.13% |
| 9 | DBP | 4 | 0.13% |
| Lifestyle behaviors | | | |
| 10 | Meat | 11 | 0.36% |
| 11 | Viscera | 11 | 0.36% |
| 12 | Vegetable | 11 | 0.36% |
| 13 | Fish | 11 | 0.36% |
| 14 | Curing food | 11 | 0.36% |
| 15 | Vegetable oil | 12 | 0.39% |
| 16 | Animal oil | 266 | 8.61% |
| 17 | Passive smoking | 0 | 0% |
| 18 | Smoking status | 0 | 0% |
| 19 | Alcohol status | 0 | 0% |
| 20 | Tea status | 0 | 0% |
| 21 | Smoking consumption | 0 | 0% |
| 22 | Wine consumption | 0 | 0% |
| 23 | Liquor consumption | 0 | 0% |
| 24 | Rice wine consumption | 0 | 0% |
| 25 | Beer consumption | 0 | 0% |
| 26 | Green tea consumption | 0 | 0% |
| 27 | Black tea consumption | 0 | 0% |
| 28 | Oolong consumption | 0 | 0% |
| Symptoms | | | |
| 29 | Chest pain | 0 | 0% |
| 30 | Anhelation | 0 | 0% |
| 31 | Palpitation | 0 | 0% |
| 32 | Cough | 0 | 0% |
| 33 | Polydipsia | 0 | 0% |
| 34 | Syncope | 0 | 0% |
| 35 | Dizziness | 0 | 0% |
| 36 | Weak | 0 | 0% |
| 37 | Urinary sign | 0 | 0% |
| 38 | Sour regurgitation | 0 | 0% |
| 39 | Stomachache | 0 | 0% |
| Medical history | | | |
| 40 | Hypertension | 0 | 0% |
| 41 | Diabetes | 0 | 0% |
| 42 | CHD | 0 | 0% |
| 43 | Arrhythmia | 0 | 0% |
| 44 | Hyperlipidemia | 0 | 0% |
| 45 | Stroke | 0 | 0% |
| 46 | Stroke of sequelae | 0 | 0% |
| 47 | Cancer | 0 | 0% |
|  | Family history |  |  |
| 48 | Family history of hypertension | 0 | 0% |
| 49 | Family history of diabetes | 0 | 0% |
| 50 | Family history of stroke | 0 | 0% |
| 51 | Family history of cancer | 0 | 0% |
| Laboratory examination | | | |
| 52 | WBC | 80 | 2.59% |
| 53 | RBC | 80 | 2.59% |
| 54 | RDWCV | 80 | 2.59% |
| 55 | MCV | 80 | 2.59% |
| 56 | MCH | 80 | 2.59% |
| 57 | MCHC | 80 | 2.59% |
| 58 | HCT | 80 | 2.59% |
| 59 | TC | 73 | 2.36% |
| 60 | TRIG | 75 | 2.43% |
| 61 | HDLc | 73 | 2.36% |
| 62 | LDLc | 137 | 4.43% |
| 63 | ALT | 73 | 2.36% |
| 64 | AST | 73 | 2.36% |
| 65 | MPV | 244 | 7.89% |
| 66 | PCT | 211 | 6.83% |
| 67 | UA | 83 | 2.69% |
| 68 | Platelet | 80 | 2.59% |
| 69 | Percentage of monocytes | 82 | 2.65% |
| 70 | Monocytes | 81 | 2.62% |
| 71 | Percentage of lymphocytes | 81 | 2.62% |
| 72 | Lymphocytes | 81 | 2.62% |
| 73 | Percentage of eosinophil | 81 | 2.62% |
| 74 | Eosinophil | 81 | 2.62% |
| 75 | Percentage of neutrophilic | 81 | 2.62% |
| 76 | Neutrophilic | 81 | 2.62% |
| 77 | Creatinine | 73 | 2.36% |
| 78 | Percentage of basophil | 80 | 2.59% |
| 79 | Basophil | 80 | 2.59% |
| 80 | Hemoglobin | 80 | 2.59% |
| 81 | Fasting glucose | 72 | 2.33% |
| ECG examination | | | |
| 82 | PWD | 165 | 5.34% |
| 83 | RV5 | 150 | 4.85% |
| 84 | SV1 | 150 | 4.85% |
| 85 | AF | 150 | 4.85% |
| 86 | Left ventricular high voltage | 150 | 4.85% |
| 87 | [PR interval](#/javascript:;) | 164 | 5.31% |
| 88 | QT interval | 151 | 4.89% |
| 89 | QTc interval | 151 | 4.89% |
| 90 | QRS interval | 150 | 4.85% |
| 91 | QRS-axis | 150 | 4.85% |
| 92 | Heart rate | 156 | 5.05% |
| 93 | Heart block | 150 | 4.85% |
| 94 | Heart transposition | 150 | 4.85% |
| 95 | ST change | 150 | 4.85% |

Abbreviations: ALT, alanine transaminase; AST, aspartate aminotransferase; AF, atrial fibrillation; BMI, body mass index; CHD, coronary heart disease; DBP, [diastolic](../../../../C:/Program%20Files%20(x86)/Youdao/Dict/8.9.9.0/resultui/html/index.html#/javascript:;) [blood](../../../../C:/Program%20Files%20(x86)/Youdao/Dict/8.9.9.0/resultui/html/index.html#/javascript:;) [pressure](../../../../C:/Program%20Files%20(x86)/Youdao/Dict/8.9.9.0/resultui/html/index.html#/javascript:;); HCT, hematocrit; HDLc, high-density lipoprotein cholesterol; LDLc, [low-density lipoprotein](../../../../C:/Program%20Files%20(x86)/Youdao/Dict/8.9.9.0/resultui/html/index.html#/javascript:;) cholesterol; MCV, mean corpuscular volume; MCH, mean corpuscular hemoglobin; MCHC, mean corpuscular hemoglobin concentration; MPV, mean platelet volume; PWD, duration of P wave; RBC, red blood cell counts; RDWCV, red blood cell volume distribution width; RV5, R wave in V5 voltage; SV1, S wave in lead V1voltage; SBP, systolic blood pressure; TG, triglycerides; TC, total cholesterol; UA, [uric](../../../../C:/Program%20Files%20(x86)/Youdao/Dict/8.9.9.0/resultui/html/index.html#/javascript:;) [acid](../../../../C:/Program%20Files%20(x86)/Youdao/Dict/8.9.9.0/resultui/html/index.html#/javascript:;); WBC, [white](../../../../C:/Program%20Files%20(x86)/Youdao/Dict/8.9.9.0/resultui/html/index.html#/javascript:;) [blood](../../../../C:/Program%20Files%20(x86)/Youdao/Dict/8.9.9.0/resultui/html/index.html#/javascript:;) [cell](../../../../C:/Program%20Files%20(x86)/Youdao/Dict/8.9.9.0/resultui/html/index.html#/javascript:;) counts；WHR, waist hip rate

**Table s3 The 95 variables and the missing rates in the Haojiang group**

| **No.** | **variable** | **Number** | **Missing rates** |
| --- | --- | --- | --- |
| Demographic characteristics | | | |
| 1 | Age | 0 | 0% |
| 2 | Gender | 0 | 0% |
| 3 | Marriage | 0 | 0% |
| 4 | Profession | 0 | 0% |
| 5 | Education | 0 | 0% |
| Physical examination | | | |
| 6 | BMI | 5 | 0.25% |
| 7 | WHR | 11 | 0.55% |
| 8 | SBP | 8 | 0.40% |
| 9 | DBP | 8 | 0.40% |
| Lifestyle behaviors | | | |
| 10 | Meat | 0 | 0% |
| 11 | Viscera | 0 | 0% |
| 12 | Vegetable | 0 | 0% |
| 13 | Fish | 0 | 0% |
| 14 | Curing food | 0 | 0% |
| 15 | Vegetable oil | 0 | 0% |
| 16 | Animal oil | 0 | 0% |
| 17 | Passive smoking | 0 | 0% |
| 18 | Smoking status | 0 | 0% |
| 19 | Alcohol status | 0 | 0% |
| 20 | Tea status | 0 | 0% |
| 21 | Smoking consumption | 0 | 0% |
| 22 | Wine consumption | 0 | 0% |
| 23 | Liquor consumption | 0 | 0% |
| 24 | Rice wine consumption | 0 | 0% |
| 25 | Beer consumption | 0 | 0% |
| 26 | Green tea consumption | 0 | 0% |
| 27 | Black tea consumption | 0 | 0% |
| 28 | Oolong consumption | 0 | 0% |
| Symptoms | | | |
| 29 | Chest pain | 0 | 0% |
| 30 | Anhelation | 0 | 0% |
| 31 | Palpitation | 0 | 0% |
| 32 | Cough | 0 | 0% |
| 33 | Polydipsia | 0 | 0% |
| 34 | Syncope | 0 | 0% |
| 35 | Dizziness | 0 | 0% |
| 36 | Weak | 0 | 0% |
| 37 | Urinary sign | 0 | 0% |
| 38 | Sour regurgitation | 0 | 0% |
| 39 | Stomachache | 0 | 0% |
| Medical history | | | |
| 40 | Hypertension | 0 | 0% |
| 41 | Diabetes | 0 | 0% |
| 42 | CHD | 0 | 0% |
| 43 | Arrhythmia | 0 | 0% |
| 44 | Hyperlipidemia | 0 | 0% |
| 45 | Stroke | 0 | 0% |
| 46 | Stroke of sequelae | 0 | 0% |
| 47 | Cancer | 0 | 0% |
| Family history | | | |
| 48 | Family history of hypertension | 0 | 0% |
| 49 | Family history of diabetes | 0 | 0% |
| 50 | Family history of stroke | 0 | 0% |
| 51 | Family history of cancer | 0 | 0% |
| Laboratory examination | | | |
| 52 | WBC | 60 | 3.01% |
| 53 | RBC | 60 | 3.01% |
| 54 | RDWCV | 60 | 3.01% |
| 55 | MCV | 60 | 3.01% |
| 56 | MCH | 60 | 3.01% |
| 57 | MCHC | 60 | 3.01% |
| 58 | HCT | 60 | 3.01% |
| 59 | TC | 52 | 2.61% |
| 60 | TRIG | 52 | 2.61% |
| 61 | HDLc | 745 | 37.36% |
| 62 | LDLc | 745 | 37.36% |
| 63 | ALT | 51 | 2.56% |
| 64 | AST | 53 | 2.66% |
| 65 | MPV | 60 | 3.01% |
| 66 | PCT | 63 | 3.16% |
| 67 | UA | 51 | 2.56% |
| 68 | Platelet | 60 | 3.01% |
| 69 | Percentage of monocytes | 752 | 37.71% |
| 70 | Monocytes | 752 | 37.71% |
| 71 | Percentage of lymphocytes | 60 | 3.01% |
| 72 | Lymphocytes | 60 | 3.01% |
| 73 | Percentage of eosinophil | 752 | 37.71% |
| 74 | Eosinophil | 752 | 37.71% |
| 75 | Percentage of neutrophilic | 60 | 3.01% |
| 76 | Neutrophilic | 60 | 3.01% |
| 77 | Creatinine | 51 | 2.56% |
| 78 | Percentage of basophil | 752 | 37.71% |
| 79 | Basophil | 752 | 37.71% |
| 80 | Hemoglobin | 60 | 3.01% |
| 81 | Fasting glucose | 51 | 2.56% |
| ECG examination | | | |
| 82 | PWD | 17 | 0.85% |
| 83 | RV5 | 1 | 0.05% |
| 84 | SV1 | 1 | 0.05% |
| 85 | AF | 0 | 0% |
| 86 | Left ventricular high voltage | 0 | 0% |
| 87 | [PR interval](#/javascript:;) | 15 | 0.75% |
| 88 | QT interval | 8 | 0.40% |
| 89 | QTc interval | 8 | 0.40% |
| 90 | QRS interval | 1 | 0.05% |
| 91 | QRS-axis | 1 | 0.05% |
| 92 | Heart rate | 1 | 0.05% |
| 93 | Heart block | 0 | 0% |
| 94 | Heart transposition | 0 | 0% |
| 95 | ST change | 0 | 0% |
|  |  |  |  |

Abbreviations: ALT, alanine transaminase; AST, aspartate aminotransferase; AF, atrial fibrillation; BMI, body mass index; CHD, coronary heart disease; DBP, [diastolic](../../../../C:/Program%20Files%20(x86)/Youdao/Dict/8.9.9.0/resultui/html/index.html#/javascript:;) [blood](../../../../C:/Program%20Files%20(x86)/Youdao/Dict/8.9.9.0/resultui/html/index.html#/javascript:;) [pressure](../../../../C:/Program%20Files%20(x86)/Youdao/Dict/8.9.9.0/resultui/html/index.html#/javascript:;); HCT, hematocrit; HDLc, high-density lipoprotein cholesterol; LDLc, [low-density lipoprotein](../../../../C:/Program%20Files%20(x86)/Youdao/Dict/8.9.9.0/resultui/html/index.html#/javascript:;) cholesterol; MCV, mean corpuscular volume; MCH, mean corpuscular hemoglobin; MCHC, mean corpuscular hemoglobin concentration; MPV, mean platelet volume; PWD, duration of P wave; RBC, red blood cell counts; RDWCV, red blood cell volume distribution width; RV5, R wave in V5 voltage; SV1, S wave in lead V1voltage; SBP, systolic blood pressure; TG, triglycerides; TC, total cholesterol; UA, [uric](../../../../C:/Program%20Files%20(x86)/Youdao/Dict/8.9.9.0/resultui/html/index.html#/javascript:;) [acid](../../../../C:/Program%20Files%20(x86)/Youdao/Dict/8.9.9.0/resultui/html/index.html#/javascript:;); WBC, [white](../../../../C:/Program%20Files%20(x86)/Youdao/Dict/8.9.9.0/resultui/html/index.html#/javascript:;) [blood](../../../../C:/Program%20Files%20(x86)/Youdao/Dict/8.9.9.0/resultui/html/index.html#/javascript:;) [cell](../../../../C:/Program%20Files%20(x86)/Youdao/Dict/8.9.9.0/resultui/html/index.html#/javascript:;) counts；WHR, waist hip rate

**Table s4 Baseline characteristic of the external validation set (N = 1994)**

| Characteristic | external validation set |
| --- | --- |
| Number | 1994 |
| End, death, n(%) | 138(6.9) |
| Time, year, median(IQR) | 2.33(2.42) |
| Age, years, median(IQR) | 68.00(7) |
| Gender, female, n(%) | 1352(67.8) |
| BMI, kg/m^2^,median(IQR) | 24.50(4.51) |
| WHR, median(IQR) | 0.92(0.08) |
| SBP, mmHg, median(IQR) | 144.50(29) |
| DBP, mmHg, median(IQR) | 86.00(16) |
| Smoking, n(%) | 449(22.5) |
| Liquor, n(%) | 24(1.2) |
| Beer, n(%) | 8(0.4) |
| Alcohol status(%) |  |
| Never | 1798(90.2) |
| Current | 132(6.6) |
| Former | 64(3.2) |
| Anhelation, n(%) | 69(3.5) |
| Sour regurgitation, n(%) | 288(14.4) |
| CHD, n(%) | 31(1.6) |
| Stroke sequelae, n(%) | 7(0.4) |
| History of cancer, n(%) | 7(0.4) |
| Family history of cancer, n(%) | 10(0.5) |
| WBC 10E+9/L, median(IQR) | 6.40(2.01) |
| [Hemoglobin, g/L, median(IQR)](#/javascript:;) | 135.00(17) |
| Percentage of monocyte(IQR) | 6.00(2.35) |
| Percentage of lymphocyte (IQR) | 36.00(10.68) |
| LDLc, mmol/L, median(IQR) | 3.69(1.23) |
| Creatinine, μmol/L, median(IQR) | 77.00(26) |
| Fasting glucose, mmol/L, median(IQR) | 5.26(1.38) |
| Heart rate, median(IQR) | 72.00(15) |
| Heart block, n(%) | 118(5.9) |
| PWD, ms, median(IQR) | 106.00(14) |
| RV5, mv, median(IQR) | 1.77(0.84) |
| SV1, mv, median(IQR) | 0.86(0.62) |

Abbreviations: BMI, body mass index; CHD, coronary heart disease; DBP, diastolic blood pressure; IQR, interquartile range; LDLc, low-density lipoprotein cholesterol; PWD, P wave duration; RV5, R wave in V5 voltage; SBP, systolic blood pressure; SV1, S wave in V1 voltage; WHR, waist hip rate; WBC, white blood cell counts

**Table s5 Effectiveness of the LASSO-Cox model**

|  | **Training set** | **Internal validation set** |
| --- | --- | --- |
| AUC(95%CI) | 0.793(95%CI 0.765-0.822) | 0.800(95%CI 0.751-0.849) |
| C-index(95%CI) | 0.792(95%CI 0.763-0.821) | 0.828(95%CI 0.785-0.871) |
| IBS(95%CI) | 0.077(95%CI 0.776-0.078) | 0.070(95%CI 0.067-0.0.073) |

Abbreviations: AUC, area under curve; C-index, concordance index; IBS, integrated Brier score

**Table s6 Nine risk factors and missing rates in the Chaonan**

|  | **Total** | | **Training set** | | **Internal validation set** | |
| --- | --- | --- | --- | --- | --- | --- |
| **Variable** | **Number** | **Missing rates** | **Number** | **Missing rates** | **Number** | **Missing rates** |
| Age | 0 | 0% | 0 | 0% | 0 | 0% |
| Gender | 0 | 0% | 0 | 0% | 0 | 0% |
| History of cancer | 0 | 0% | 0 | 0% | 0 | 0% |
| Alcohol status | 0 | 0% | 0 | 0% | 0 | 0% |
| Daily liquor consumption | 0 | 0% | 0 | 0% | 0 | 0% |
| Hemoglobin | 80 | 2.59% | 16 | 2.72% | 64 | 2.76% |
| Fasting glucose | 72 | 2.33% | 21 | 2.07% | 51 | 2.20% |
| Heart rate | 156 | 5.05% | 36 | 4.66% | 120 | 5.18% |
| Heart block | 0 | 0% | 0 | 0.00 | 0 | 0% |

**Table s7 Comparison of predictive capabilities of different algorithms**

| **Algorithms** | **Training set** | **Internal validation set** |
| --- | --- | --- |
| LASSO, AUC(95%CI) | 0.783(95%CI 0.754-0.813) | 0.789(95% CI 0.740-0.838) |
| Elastic net, AUC(95%CI) | 0.787(95% CI 0.758-0.816) | 0.791(95% CI 0.741-0.841) |
| XGBoost, AUC(95%CI) | 1.000(95% CI 1.000-1.000) | 0.613(95% CI 0.596-0.678) |

**Table s8 Comparison of predictive capabilities of different models**

| **Models** | **Training set** | **Internal validation set** | **External validation set** |
| --- | --- | --- | --- |
| **AUC(95%CI)** | | | |
| Nomogram on elastic net-Cox | 0.768(95%CI 0.737-0.798) | 0.784(95%CI 0.736-0.832) | 0.770(95%CI 0.728-0.811) |
| Nomogram on LASSO-Cox | 0.767(95%CI 0.736-0.798) | 0.776(95%CI 0.722-0.831) | 0.806(95%CI 0.768-0.845) |
| Nomogram on age and sex | 0.734(95%CI 0.701-0.768) | 0.745(95%CI 0.693-0.798) | 0.750(95%CI 0.704-0.797) |
| **C-index****(95%CI)** | | | |
| Nomogram on elastic net-Cox | 0.752(95%CI 0.719-0.785) | 0.777(95%CI 0.726-0.828) | 0.769(95%CI 0.731-0.807) |
| Nomogram on LASSO-Cox | 0.765(95%CI 0.733-0.797) | 0.775(95%CI 0.719-0.831) | 0.797(95%CI 0.76-0.834) |
| Nomogram on age and sex | 0.714(95%CI 0.678-0.749) | 0.732(95%CI 0.676-0.788) | 0.745(95%CI 0.701-0.79) |
| **IBS(95%CI)** | | | |
| Nomogram on elastic net-Cox | 0.081(95%CI 0.026-0.136) | 0.076(95%CI 0.033-0.12) | 0.049(95%CI 0.018-0.081) |
| Nomogram on LASSO-Cox | 0.079(95%CI 0.037-0.123) | 0.076(95%CI 0.045-0.108) | 0.047(95%CI 0.012-0.083) |
| Nomogram on age and sex | 0.082(95%CI 0.028-0.137) | 0.080(95%CI 0.042-0.117) | 0.050(95%CI 0.021-0.08) |

Abbreviations: AUC, area under curve; C-index, concordance index; IBS, integrated Brier score

**
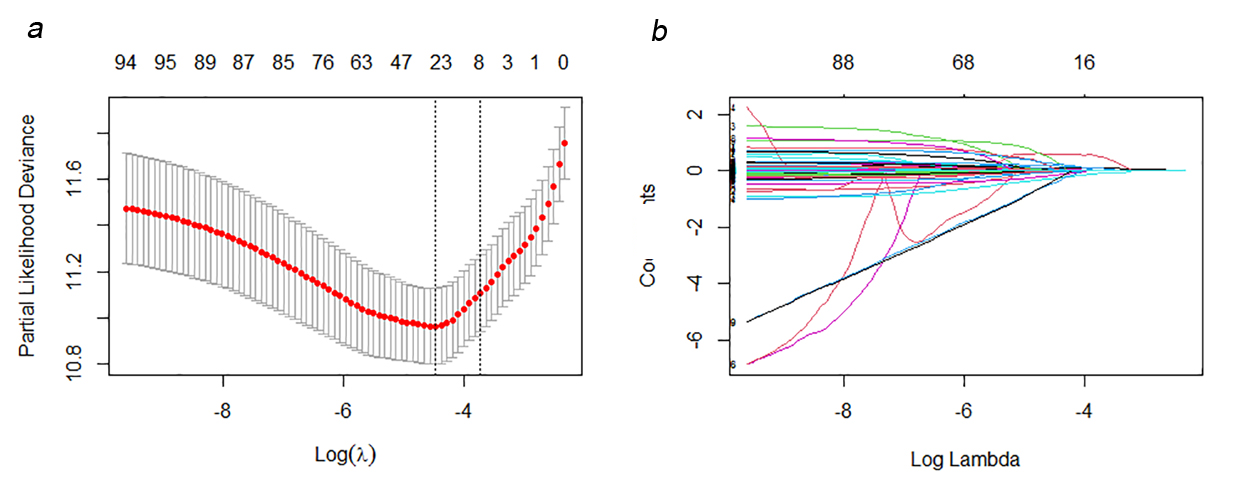
**

**Fig s1**


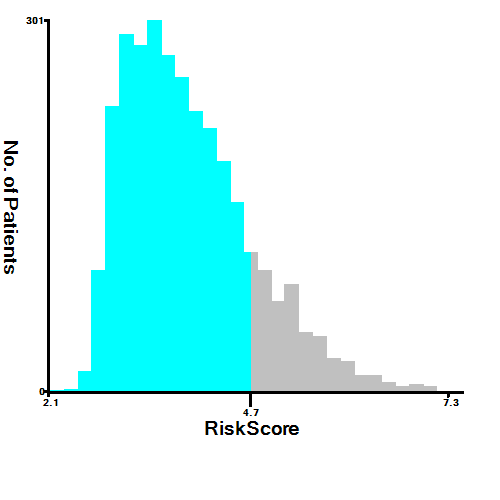
**Fig s2**


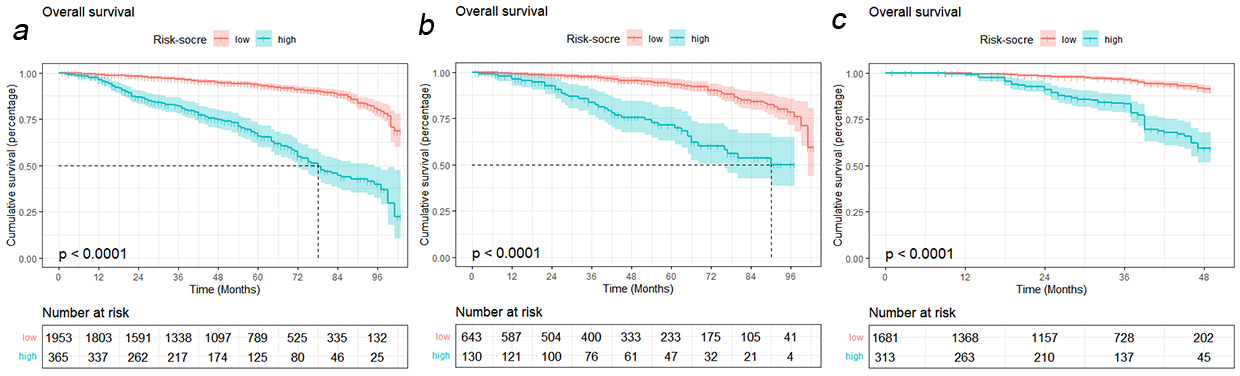
**Fig s3**
