# Supplementary material for: Predicting 1-, 3-, 5-, and 8-year all-cause mortality in a community-dwelling older adult cohort: relevance for predictive, preventive, and personalized medicine
Source: EPMA J. 2023 Nov 3;14(4):713–26. doi: 10.1007/s13167-023-00342-4 (PMC10713970; doi:10.1007/s13167-023-00342-4)
Supplement: Supplementary file 2 — Supplementary file2 (DOC 38 KB) [file 13167_2023_342_MOESM2_ESM.doc]

**PPPM Innovation Highlights**

**A: Working hypothesis**

It is hypothesized that an early application PPPM approach would contribute to the healthcare of older adults. Compared to a separate questionnaire, the clinical multidimensional variables add an electrocardiogram (ECG) and clinical laboratory examination, which reflect the characteristics of the participants more comprehensively and objectively. In this study, we predict that an accurate all-cause mortality risk prediction model can be developed using clinical multidimensional variables for community-dwelling older adults. Such a model would add value to personalized medicine for community-dwelling older adults by enabling PPPM. To test our hypothesis, we developed and externally validated a nomogram prediction model. AUC, C-index, IBS and calibration curves were used to evaluate the discriminatory and calibration abilities of the nomogram. Decision curve analysis was used to assess the clinical utility of the nomogram.

**B：Innovation towards the**

**1. predictive approach,**

**2. targeted prevention and**

**3. personalization of medical services.**

This is the first study using clinical multidimensional variables to develop and validate a nomogram prediction model for 1-, 3-, 5-, and 8-year all-cause mortality among community-dwelling older adults with external validation. Our study supports the application of PPPM, from different perspectives, in the medical services of all-cause mortality community-dwelling older adults. Firstly, from the perspective of all-cause mortality prediction, we constructed an all-cause mortality risk prediction model applicable to community-dwelling older adults. The risk prediction model could identify individuals at high risk of all-cause mortality and contribute to the paradigm shift from delayed reactive medicine to proactive medicine, which could help save public health resources and decrease the mortality of community-dwelling older adults. Secondly, from the perspective of all-cause mortality prevention, currently the prediction indicators of mortality prediction models are limited in demographic variables, comorbid conditions and lifestyle behaviors. In this study, we identified risk factors by clinical multidimensional variables, and these risk factors are easy to obtain, thus helping us to monitor the progression of all-cause mortality. Thirdly, from the perspective of personalized interventions, clinical multidimensional variables are more comprehensive and objective. Our risk prediction model can predict the risk of mortality for community-dwelling older adults at different times, and develop tailored interventions through modifiable risk factors to reduce mortality.

1. **How does the presented innovation go beyond the state of the art contributing to the paradigm shift from reactive medicine to PPPM?**

Prior research mainly focuses on exploring the progress and prognosis of the disease to reduce mortality . However, few studies have predicted all-cause mortality in community-dwelling older adults. For these hospitalized elderly patients who have significant symptoms, the current health care outcomes are considered inadequate. Compared with previous reactive medicine, PPPM is a new integrative paradigm that focuses on predicting and preventing disease before the onset of symptoms, as well as providing personalized treatment, and focuses on proactive preventative measures . All-cause mortality prediction, targeted prevention and intervention before the onset of symptoms is important, and could improve the quality of life and extend life expectancy. Previous all-cause mortality prediction models for community populations have been developed in Europe, but their prediction indicators are limited in demographic variables, comorbid conditions and lifestyle behaviors . The participants of these studies were not limited to community-dwelling older adults, and also included young and middle-aged adults, and none of these models underwent external validation . There is especially a lack of studies to predict all-cause mortality for community-dwelling older adults in China. To solve this problem, we developed an all-cause mortality risk prediction model in community-dwelling older adults to identify individuals on the basis of individual heterogeneity following the concept of PPPM. To the best of our knowledge, this is the first study using clinical multidimensional variables to develop and validate a nomogram prediction model, with external validation, for 1-, 3-, 5-, and 8-year all-cause mortality among community-dwelling older adults. Our study fills a gap, in the framework of PPPM, for all-cause mortality prediction with multidimensional data and external validation to provide targets for proactive prevention and individual management in personalized medicine of community-dwelling older adults. This easy-to-use prediction model can easily identify elderly people at high-risk of all-cause mortality, guide targeted prevention, provide personalized interventions for high-risk individuals, and promote a paradigm shift from delayed reactive medicine to proactive medicine. (See Page 8)

**References**

[7] Golubnitschaja O, Kinkorova J, Costigliola V. Predictive, Preventive and Personalised Medicine as the hardcore of 'Horizon 2020': EPMA position paper. *Epma j*. 2014;**5**:6. doi: 10.1186/1878-5085-5-6.

[25] Zhou Y, Chen Y, Zhang X, Zhao B, Gao F, Yuan X, et al. Nutritional risk and a high NRS2002 score are closely related to disease progression and poor prognosis in patients with COVID-19. *Frontiers in nutrition*. 2023;**10**:1089972. doi: 10.3389/fnut.2023.1089972.

[26] Ding D, Rogers K, van der Ploeg H, Stamatakis E, Bauman AE. Traditional and Emerging Lifestyle Risk Behaviors and All-Cause Mortality in Middle-Aged and Older Adults: Evidence from a Large Population-Based Australian Cohort. *PLoS medicine*. 2015;**12**:e1001917. doi: 10.1371/journal.pmed.1001917.

[27] Fleischmann KE, Orav EJ, Lamas GA, Mangione CM, Schron E, Lee KL, et al. Pacemaker implantation and quality of life in the Mode Selection Trial (MOST). *Heart rhythm*. 2006;**3**:653-9. doi: 10.1016/j.hrthm.2006.02.1031.

[28] Kobayashi LC, Jackson SE, Lee SJ, Wardle J, Steptoe A. The development and validation of an index to predict 10-year mortality risk in a longitudinal cohort of older English adults. *Age and ageing*. 2017;**46**:427-32. doi: 10.1093/ageing/afw199.

[29] B ÓH, Gransar H, Callister T, Shaw LJ, Schulman-Marcus J, Stuijfzand WJ, et al. Development and Validation of a Simple-to-Use Nomogram for Predicting 5-, 10-, and 15-Year Survival in Asymptomatic Adults Undergoing Coronary Artery Calcium Scoring. *JACC Cardiovascular imaging*. 2018;**11**:450-8. doi: 10.1016/j.jcmg.2017.03.018.
